# Discussing Different Approaches for the Time-Zero as Start for Autogenous Shrinkage in Cement Pastes Containing Superabsorbent Polymers

**DOI:** 10.3390/ma12182962

**Published:** 2019-09-12

**Authors:** José Roberto Tenório Filho, Maria Adelaide Pereira Gomes de Araújo, Didier Snoeck, Nele De Belie

**Affiliations:** 1 Magnel Laboratory for Concrete Research, Department of Structural Engineering, Faculty of Engineering and Architecture, Ghent University, B-9052 Ghent, Belgium; 2 SIM vzw, Technologiepark 48, Zwijnaarde, B-9052 Ghent, Belgium; 3 Polymer Chemistry and Biomaterials Group, Department of Organic and Macromolecular Chemistry, Ghent University, B-9052 Ghent, Belgium

**Keywords:** autogenous shrinkage, time-zero, superabsorbent polymers

## Abstract

Many studies have already been published concerning autogenous shrinkage in cementitious materials. Still, no consensus can be found in the literature regarding the determination of the time-zero to initiate the recording of autogenous shrinkage. With internal curing agents, a correct evaluation of their efficiency depends on an appropriate choice of the time-zero. This study investigates different approaches to estimate the time-zero for cement paste mixtures with and without superabsorbent polymers as internal curing agents. The initial and final setting times were determined by an electronic Vicat and ultrasonic pulse velocity measurements (UPV); the transition point between the fluid and solid state was determined from the autogenous strain curve; the development of the capillary pressure was also studied. The choice of time-zero before the transition point led to higher values of shrinkage strain that should not be taken into account for autogenous shrinkage. A negligible difference was found between the strains when the final setting time and the transition point were taken as time-zero. Considering the artefacts and practical issues involving the different methods, the use of the transition point from the autogenous strain curve is the most suitable technique for determining the time-zero.

## 1. Introduction

In cementitious materials, after the contact of water with the cement and during hydration, many changes take place in the material’s structure. From that moment until the cementitious material reaches its final setting, chemical and physical processes result in expansion and shrinkage that can initiate cracking in the hardening material. 

Shrinkage in concrete structures has been the focus of many studies, and lately a lot of attention has been given to autogenous shrinkage. Although the autogenous shrinkage may not be prominent in ordinary concrete structures, in systems with water-to-cement or to-binder ratios lower than 0.42 (ultra-high performance concrete, for example) it can become a serious issue associated with the cracking of the structure at early age [1,2]. 

In the first moments of cement hydration, a large volume change is noticed mainly due to chemical shrinkage. Since it happens during a stage at which the cementitious material is still very fluid there are no major concerns regarding the risk of cracking. In this context, some authors suggest that the measurements of autogenous shrinkage should start at the moment where the material is able to resist the tensile stresses which occur right after the transition of the material from a liquid to a solid state [3,4,5,6]. This moment in time is referred to as time-zero. 

While there is a certain agreement in the definition, there is still a lack of consensus on how to determine that point and many different techniques have been reported: the initial setting time by means of the Vicat test [7,8,9]; the final setting time by means of the Vicat test [5]; the final setting time by means of ultrasonic pulse velocity (UPV) [10,11,12,13]; the maximum negative rate of electric conductivity [4]; the knee point observed in autogenous shrinkage measurements by means of corrugated tubes [14]. In some codes and studies, even some arbitrary values of time are used. For example, in the case of ASTM C157 [15], an indication of 24 h is used. 

As it can be seen, a lot of different methods and techniques have been reported. However, not all of them represent the same phenomenon and some artefacts related to the test methods may hinder a comparative analysis, the choice of the best technique, and ultimately the study and conclusion on materials used to mitigate shrinkage. 

Among the group of techniques based on the solidification of the material, the Vicat test seems to be the most used one given its simple approach. However, despite being simple to perform, the method is based on arbitrary thresholds to define the time of set. The ASTM C191 [16], for instance, defines the initial setting time as the time when a penetration of 25 mm is obtained, and the final setting time as the time when the needle does not sink visibly into the paste. The EN 196–3 [17], on the other hand, defines the initial setting time as the moment at which the distance between the needle and the base-plate is (6 ± 3) mm and the final setting time as the moment at which the needle first penetrates only 0.5 mm into the specimen. Some standards also require the setup to be covered (NBN EN 13294 [18]), while others, as mentioned above, are exposed to the air, influencing the setting of the cementitious material. 

The UPV method is more related to the development of the microstructure of the material and should be more accurate than the Vicat method [19,20]. 

As for the conductivity measurements, despite the fact that a lot of studies have been developed in the field, it is still questionable if it is possible to identify the time-zero based on this method. While it might be useful to obtain information on the chemical processes during cement hydration, the method itself does not directly reflect the physical changes in the material [21,22].

Autogenous shrinkage is known to be related to a reduction in the internal relative humidity of the material (a consequence of the cement hydration) and associated to the development of the capillary pressure in the pore system due to the receding menisci [1,23,24]. Based on this understanding, some other approaches were established making use of different techniques, such as: the capillary pressure monitoring to assess the time-zero [25,26]; the divergence point between the curves of chemical shrinkage and autogenous strain [3,27]; the drop in relative humidity [28,29]; and the rate of autogenous strain [3,30]. All those methods are more directly related to the physical mechanisms behind the autogenous shrinkage. 

Being able to choose an appropriate time-zero value is of utmost importance since it might have a major impact on the interpretation of the shrinkage values which could lead to misinterpretations of results such as an underestimation of strain or overestimation of the effect of internal curing/shrinkage reducing agents.

The use of internal curing agents has proven to be quite effective in the mitigation of autogenous shrinkage [31,32,33,34] and special attention has been given to the use of superabsorbent polymers (SAPs) [28,35,36,37,38,39,40,41,42].

Superabsorbent polymers (or hydrogels) are a natural or synthetic water-insoluble 3D network of polymeric chains cross-linked by chemical or physical bonding. They possess the ability to take up a significant amount of fluids from the environment (in amounts up to 500 times their own weight) [35]. 

Once in contact with the mixing water of the cementitious material, the SAPs absorb and retain a certain amount of the water (depending on their absorption capacity), later on acting as water reservoirs for the system, keeping its levels of internal relative humidity high for a considerable time frame [37,40,43]. The swelling and the posterior water release are of great interest in the study of smart self-healing materials, but can also be explored to promote self-sealing [35,37,41,44]. 

As the internal curing takes place and more water is provided to the system, the time for the start of the autogenous shrinkage is expected to change in comparison to mixtures without this feature. This also changes the behavior of the material when tested with the different methods cited above. 

This study examines different techniques to determine the time-zero for the autogenous shrinkage measurements in cement pastes with and without SAPs, exploring the influence of the different methods on the final values of the autogenous strain. The suitability of the different methods is discussed for the different paste compositions and a recommendation is provided especially for the case of SAPs. In this way, a more uniform testing procedure will allow to further investigate the materials and to optimize them in practical applications. Based on these conclusions, the influence of the SAPs in terms of time zero is discussed, to fill the gap in literature.

## 2. Materials and Methods 

The experimental program was based on the measurement of the autogenous shrinkage deformation of the mixtures. The hardening of the mixtures was studied by means of the Vicat test and UPV. The build-up in the capillary pressure was monitored and the rate of deformation due to the autogenous strain was used to determine the transition point between the fluid and solid state of the mixtures. An air void analysis was also performed. 

Prior to all the tests, the SAPs were characterized in terms of their water uptake in cement paste. Their absorption capacity was measured by comparing the flow table values of the cement paste mixtures (according to the method described in the EN 1015–3 [45]) exactly 10 min after the first contact of the dry SAPs with the water in the paste.

In total, four mixtures were studied, two without and two with SAPs. All tests were performed in a room with controlled temperature (20 ± 2 °C) and relative humidity (60 ± 5%).

### 2.1. Mixture Compositions 

All tests were performed on cement pastes produced with cement type CEM III-B 42.5N—LH/SR (CBR, Zeebrugge, Belgium); a polycarboxylate superplasticizer (at a constant dosage of 0.3 m% in relation to the cement mass; Glenium 51, 35% conc., BASF, Oosterhout, The Netherlands); and two superabsorbent polymers hereby identified as SAP1 and SAP2, which will be discussed below. 

SAP1, provided by BASF, Trostberg, Germany, is a copolymer of acrylamide and sodium acrylate produced by bulk polymerization and subsequently ground to a mean particle size (D50) of 40 μm. It has an absorption capacity of 27 g/g (grams of mixing water per grams of SAPs). SAP2, produced by SNF Floerger, Andrézieux-Bouthéon, France, is a cross-linked acrylate copolymer also produced by bulk polymerization and a mean particle size (D50) of 360 μm with absorption capacity of 21 g/g (grams of mixing water per grams of SAPs). 

The mixing procedure adopted for all the mixtures followed the standard NBN EN 196–1 [46] with the superplasticizer added after the water. For the SAP-containing mixtures, the SAPs were added together with the dry materials and dry mixed before the addition of water to ensure a uniform distribution of the SAPs.

Information on the composition of the cement pastes can be found in Table 1. SAPs and superplasticizer are added as determined by mass of cement weight (m%). The amount of superplasticizer was kept constant, to minimize the effect on the setting of the materials with and without SAPs [39].

A water-to-cement ratio of 0.30 was chosen, as this REF0.3 mixture would show pronounced autogenous shrinkage [38]. The second reference, REF0.354 was included based on the theory of Powers and Brownyard [47] which was adapted by Jensen and Hansen [1] for the case of internal curing. According to their study, an additional amount of water corresponding to an entrained water-to-cement ratio of 0.054 stored in the SAPs is enough for an effective internal curing in mixtures produced with ordinary Portland cement and a water-to-cement ratio of 0.30. The amount of SAPs for mixtures SAP1 and SAP2 was chosen as such that the additional entrained water (0.054) would be absorbed (based on their absorption capacity in cement paste). These amounts were also found to effectively mitigate the autogenous shrinkage of the cement pastes [42].

### 2.2. Measuring the Autogenous Shrinkage 

The autogenous shrinkage was assessed according to the standard ASTM C1698–09 [48]. The test consists of the measurement of the deformation of cement pastes in corrugated tubes with a nominal length of 425 ± 5 mm and a diameter of 29 ± 0.5 mm. The specimens were placed on metallic supports with one linear variable differential transducer (LVDT) with a range of 5 mm on one end. The other end was restrained in movement. The measurements were performed continuously every 10 min for 7 days in a controlled atmosphere of 20 ± 2 °C and 60 ± 5% RH. Even though the temperature variation inside the specimen might influence its shrinkage behavior, a previous study where tubes were immersed in a polyalkylene-glycol thermobath at 20 °C and in parallel placed on a manual measuring bench in a temperature-controlled room, concluded that the increase in temperature did not have a significant effect and all results were comparable [38].

### 2.3. Setting and Hardening of the Mixtures

The setting and hardening of the mixtures were studied by means of an electronic Vicat apparatus in compliance with EN 196–3 [17]. The specimen used for the Vicat was a truncated cone with a diameter of 80 mm at the base, 70 mm at the top and height of 40 mm. During the test, the specimen was exposed to the room environment (i.e., 20 ± 2 °C and 60 ± 5% RH).

The UPV was determined with a FreshCon equipment [11] (Smartmote, Stuttgart, Germany) compressive pulse waves. The measurements were automatically performed each 5 min during 24 h with an amplifying voltage of 450 V and a pulse signal with a width of 2.5 µs. A U-shaped specimen with approximately 35 cm^3^ of volume was used. The distance between the sender and receiver point was 22 mm. During the measurements, the specimen was covered by plastic foil connected to the mold by a thin layer of Vaseline to prevent drying shrinkage.

### 2.4. Build-Up in the Capillary Pressure

**Figure 1:**
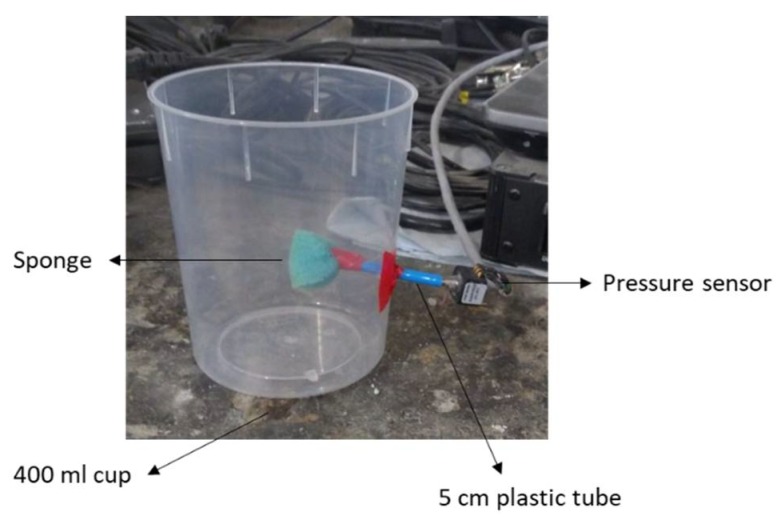
Setup used for the capillary pressure monitoring.

A small pressure transducer (RVAP015GU, Sensortronics, Puchheim, Germany) registered the development of the capillary pressure every 10 min for 24 h. The custom-made test setup is shown in Figure 1. In the test, a 400 mL plastic cup is filled with the cement paste. At a height of 37 mm from the bottom, a plastic tube of 50 mm length is inserted in the cup. At the inner end of the tube a piece of sponge is attached while the other end is connected to the pressure transducer. Once water migrates from the cement paste to the tube, the transducer registers the variance in the voltage. The sponge acts as a preliminary filter that prevents the cement paste from coming into the tube. Right after filling, the cup was closed with a plastic lid to prevent drying shrinkage.

### 2.5. Air Void Analysis 

Small plate specimens (50 × 50 × 20 mm) were prepared from the same mixtures used for the other tests. After 28 days of curing those specimens were polished and prepared for an air void analysis with a RapidAir image analysis system (Germann Instruments, Copenhagen, Denmark), in accordance to the EN 480–11 [49].

## 3. Results and Discussion

In this part, the results will be discussed, first the setting time (3.1) will be investigated, followed by the transition point between fluid and solid state based on the rate of autogenous strain, determined with the corrugated tubes (3.2) and the development of capillary pressure (3.3). Following that, the autogenous strains for different time-zeros are presented (3.4) and the section is closed with a discussion about practical issues of the different test methods (3.5).

### 3.1. Setting Time

**Figure 2:**
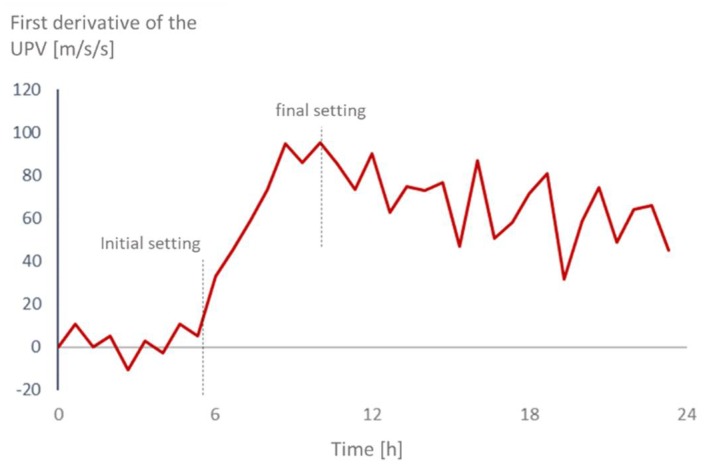
Determining the initial and final setting point based on the rate of the UPV (REF0.3). In order to improve the visualization of the graph, values are averaged over 30 min.

Both the initial and the final setting times with the Vicat test were determined based on the thresholds described by the Standard EN 196–3 [17]. With the UPV, the values were determined based on the rate of the velocity. The typical curve for the wave velocity, as it has been found in many studies [10,11,12,13,37,50,51,52,53], is marked by two points where there is a sudden increase and then a reduction in the rate of the UPV. These points are very similar to the ones found during the transition of periods between the induction and acceleration phases of the cement hydration. They can be easily identified by plotting the first derivative of the UPV over time (Figure 2), where the point right before the sudden increase in the velocity rate is taken as the initial setting time and the maximum value of the velocity rate is taken as the final setting point. 

The initial and final setting times determined using the above-mentioned test methods are shown in Table 2.

**Figure 3:**
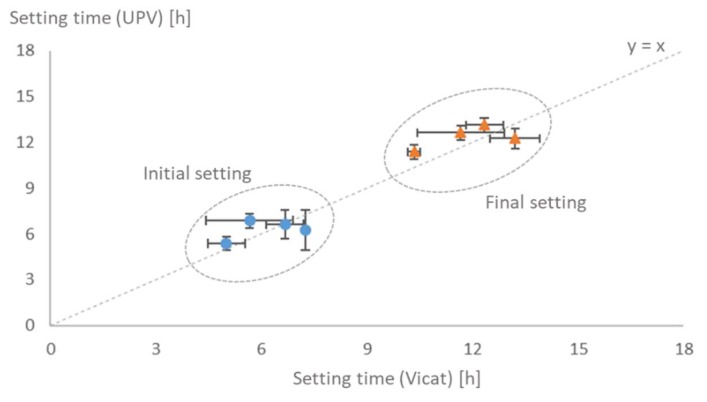
Comparison between setting times determined by Vicat and UPV methods.

Despite the differences between the test procedures, the specimens’ size and the physical aspects of the test procedures, the results present considerable correspondence, as shown in Figure 3.

Comparing the standard deviations of the test methods, the UPV measurements seem to give more consistent results in terms of final setting time. This may be due to the exposure of the specimens to the environment in the case of the Vicat measurements, which increases the effects of the drying shrinkage. In this way, the height of the specimens varies in a wider range during the test, which hinders the value to be taken as the threshold for the final setting time.

The increase in water-to-cement ratio for the mixture without SAPs (i.e., REF0.354) caused a small delay in both the initial and final setting time. As for the mixtures containing SAPs, the initial setting times were somewhere in between the original reference (REF0.3) and the reference containing the same amount of total water as the mixtures with SAPs (REF0.354). This result can be explained by the gradual water release of the SAPs that put the mixtures in an intermediate state with regards to water content before the final setting occurs. 

It can be also noted that, for the mixture SAP2, the final setting time determined by the UPV happens slightly later compared to the time measured for the reference mixtures and the mixture SAP1, while this was not observed with Vicat test. This indicates that the UPV method is more sensitive to the microstructure of the material than the Vicat method, since SAP2 has bigger particles than SAP1, thus an expected higher porosity. In fact, an air void analysis showed an amount of air content of 4.76% for SAP2 (with 1.57% for voids in the range between 505–1000 microns, 0.99% in the range between 1005–1500 microns, and 0.13% in the range between 1505–2000 microns) and 2.59% for SAP1 (with only 0.073% of voids in the range between 505–1000 microns, being this the highest void size range of the mixture).

### 3.2. Transition Point between Fluid and Solid State Based on the Rate of Autogenous Strain

**Figure 4:**
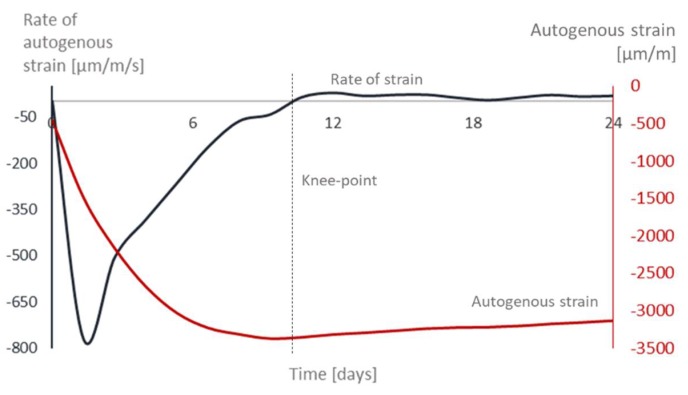
Determining the knee-point of REF0.354.

The transition point between solid and fluid state was determined considering the moment where the rate of autogenous strain becomes zero. From that moment onwards, in the autogenous strain curve a change in the deformation of the material where the strain rate suddenly and temporarily shifts to a more constant behavior is noticed (Figure 4). 

The results for all the tested mixtures are presented in Table 3. When comparing with the results obtained with the Vicat and UPV methods, the knee-point occurs closer to the final setting for the mixtures. 

**Figure 5:**
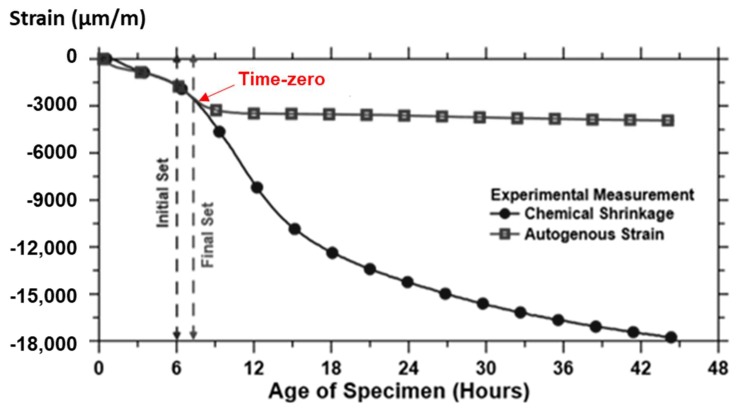
Deviation between chemical shrinkage and autogenous strain as a procedure to determine time-zero. Adapted from [4]. The permission to reuse the figure was granted by RILEM Publications on September 9, 2019.

Previous research [3] showed also a correlation between the final setting time determined by the Vicat apparatus and the divergence point between the chemical and autogenous shrinkage curves (occurring around the final setting time, as shown in Figure 5), also identified as the point where the rate of autogenous strain becomes closer to zero. According to the authors, while the Vicat test is relatively simple to perform, it uses an arbitrary method to define the time of set and this should be taken into account when choosing a suitable method to determine the time-zero.

Sant et al. [4] also found a delay of 1.5–2 h in the final setting time determined by the Vicat method of cement paste mixtures containing shrinkage reducing admixtures (SRA) in relation to reference mixtures without the SRA’s. The same trend was observed when comparing the transition points of the autogenous strain curves.

### 3.3. Development of Capillary Pressure

**Figure 6:**
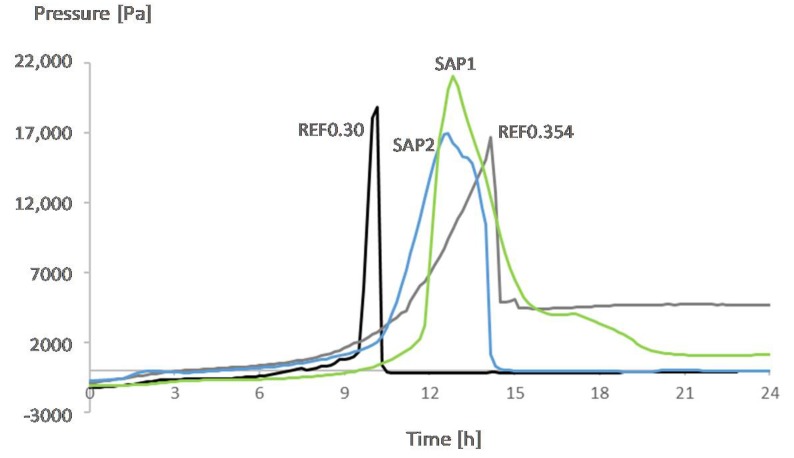
Development of the capillary pressure over time for all mixtures.

Figure 6 shows the capillary pressure values over time for all mixtures. In comparison with the mixture REF0.3, all the other mixtures present a small delay in the time for the highest build-up in the pressure. This points to the presence of more water (REF0.354) or to the gradual and continued water release by the SAPs for internal curing purposes. 

The structure of the capillary network at very early age should be mostly determined by the amount of water in the mixture [25]. Inside the material, the water consumption is expected to occur first in the bigger pores and afterwards in the smaller ones. Since REF0.354 has the highest effective water-to-cement ratio among all the mixtures, a higher volume of bigger pores in comparison to the other mixtures is expected. An air void analysis showed that for the mixture REF0.3 the highest void size is in the range between 355–400 microns, while for the mixture REF0.354 this value is in the range between 455–500 microns. The water consumption in such pores is slower than in the smaller pores which then leads to a delay in the pressure build-up. Also, the higher amount of water might have led to a partial emptying of the pores right after the pressure break-down, leading to a higher constant value of pressure for REF0.354 in comparison to the other mixtures after 15 h.

By increasing the water-to-cement ratio in the mixture REF0.354 in comparison to the mixture REF0.3, a change in the maximum pressures is noticed. The higher water-to-cement ratio reduces the value of the maximum pressure but at the same time delays the time when such value is reached. These findings are in accordance with the literature [26].

In mixtures SAP1 and SAP2, since the water release by the SAPs starts to occur once the water is being consumed inside the pores of the material and occurs gradually, the moment of pressure build-up is somewhere between REF0.3 and REF0.354.

Duplicate specimens were tested for all the mixtures and the highest difference in the time for building-up in the capillary pressure was 1.17 h (for mixture SAP1).

### 3.4. Autogenous Strain for Different Time-Zeros

**Figure 7:**
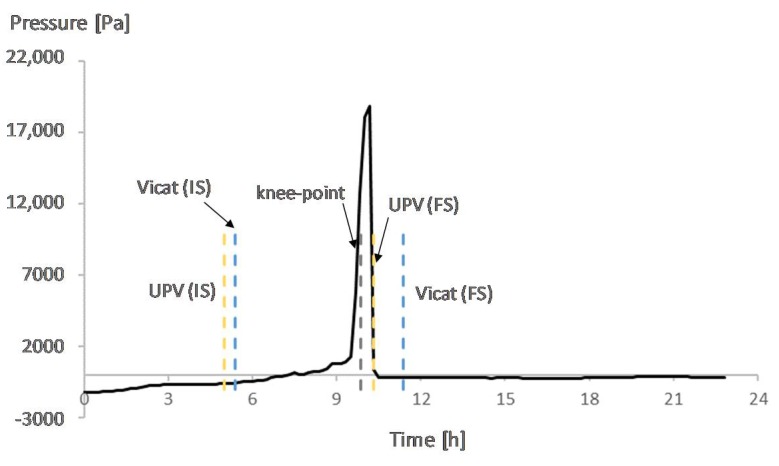
Capillary pressure, setting times, and transition point of REF0.3. With IS referring to initial setting and FS referring to final setting.

**Figure 8:**
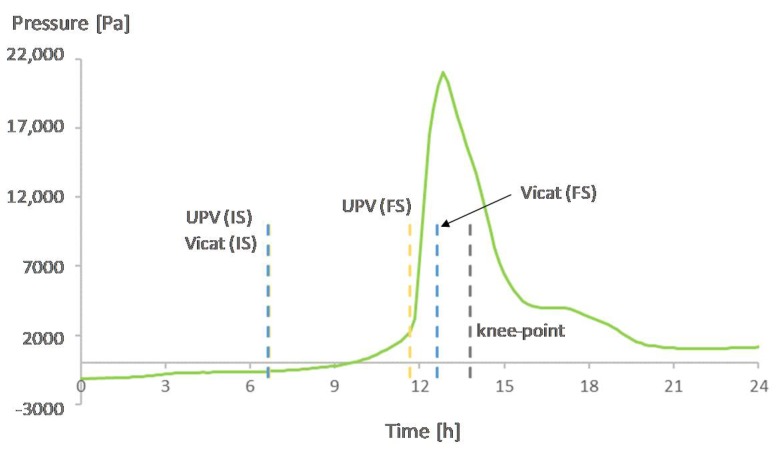
Capillary pressure, setting times, and transition point of SAP1. With IS referring to initial setting and FS referring to final setting.

**Figure 9:**
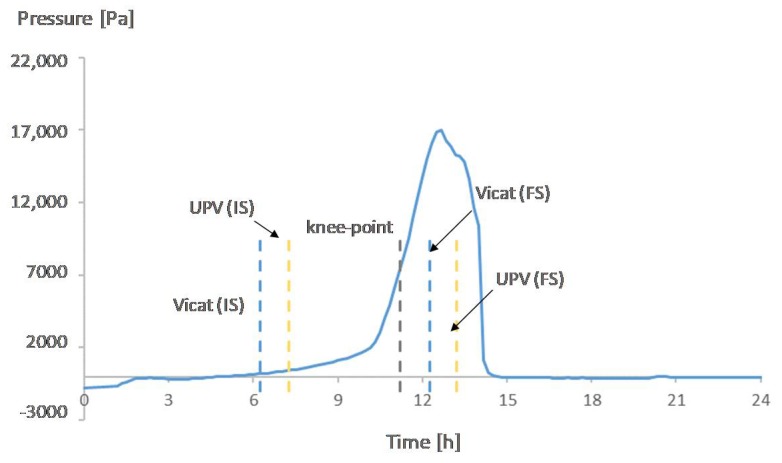
Capillary pressure, setting times, and transition point of SAP2. With IS referring to initial setting and FS referring to final setting.

**Figure 10:**
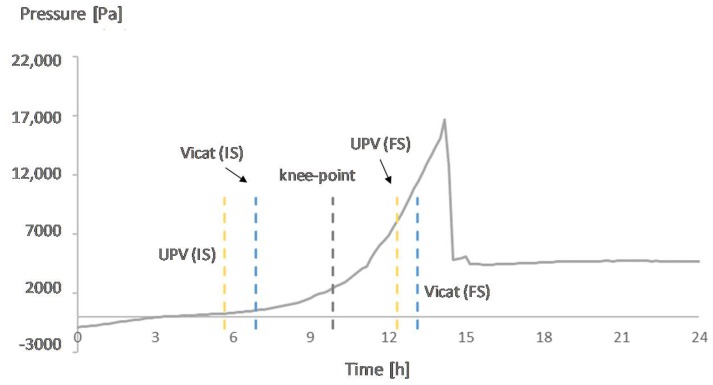
Capillary pressure, setting times, and transition point of REF0.354. With IS referring to initial setting and FS referring to final setting.

Figure 7, Figure 8, Figure 9 and Figure 10 show the development of the capillary pressure for each mixture with the indication of the initial and final setting time (determined by both the Vicat and UPV methods) and the transition time determined by the rate of autogenous strain.

For all mixtures, except for REF0.354, the build-up in capillary pressure occurs near the knee-point and the final setting time (Figure 7, Figure 8 and Figure 9), with a negligible difference considering the standard deviations (Table 2 and Table 3). This shows a correspondence between the solidification moments and the development of higher stresses inside the pores.

For the mixture REF0.354, the transition point occurs before the final setting time. The pressure starts to build-up around the knee-point and reaches its break-down after the final setting (Figure 10). In comparison with the mixture REF0.3, due to the higher amount of water, the pores formed after the transition from liquid to solid state take longer to be emptied. Also, the formation of a stable solid skeleton is an essential condition for the self-desiccation, but it does not necessarily mean that this phenomenon will take place immediately after the formation of the skeleton [25], which explains why the break-down in pressure occurs only a few hours after the knee-point. Tests performed with a reference mixture produced with a water-to-cement ratio of 0.4 showed the same trend in behavior: the moment for the maximum build-up in the capillary pressure occurred around 3 h later than in the REF0.354, which was closer to the time of final setting determined with the Vicat method than to the knee-point. 

**Figure 11:**
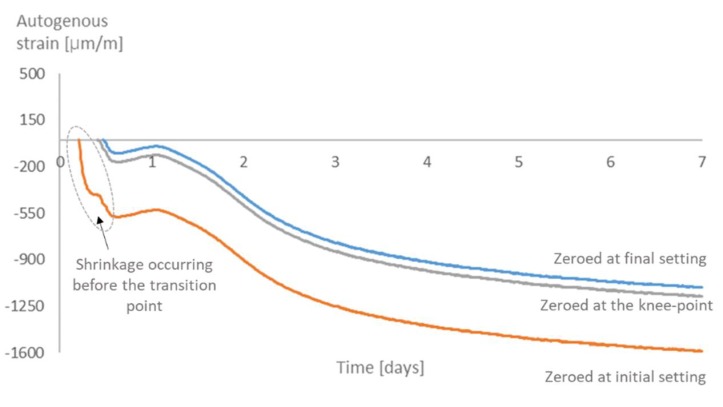
Autogenous strain for different time-zeros in REF0.3.

**Figure 12:**
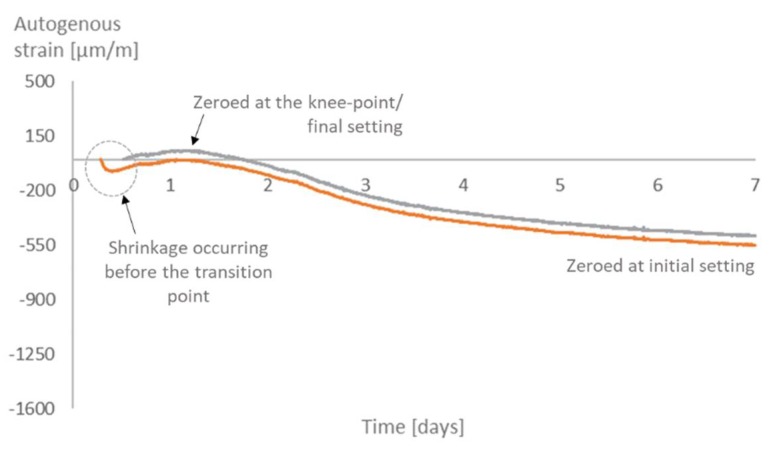
Autogenous strain for different time-zeros in REF0.354.

**Figure 13:**
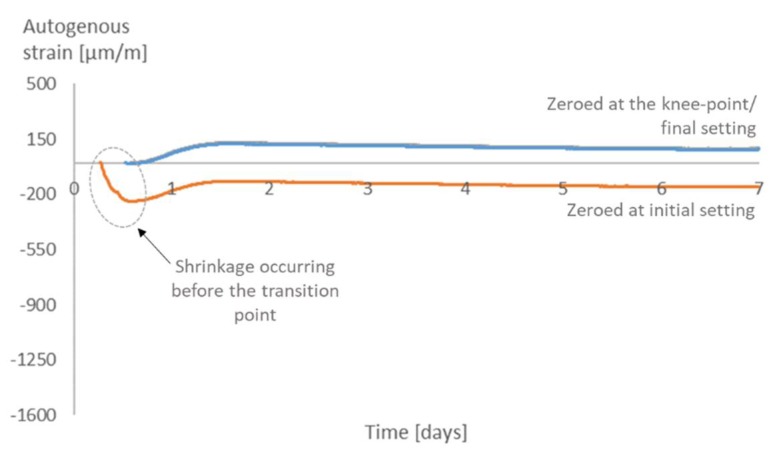
Autogenous strain for different time-zeros in SAP1.

**Figure 14:**
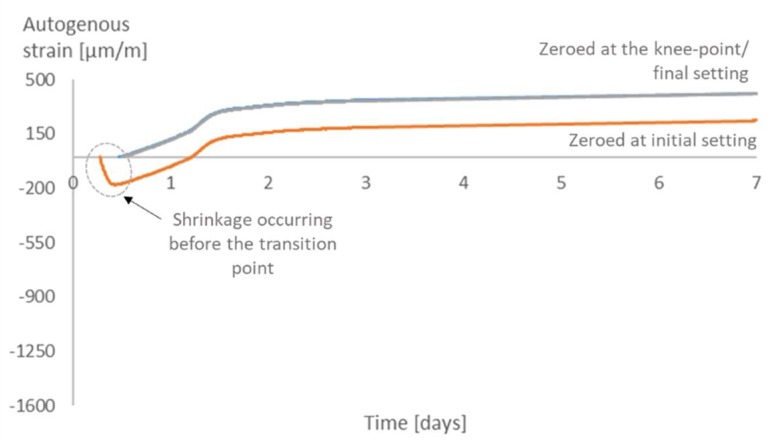
Autogenous strain for different time-zeros in SAP2.

As for the time-zero, for all mixtures, there is not much difference in the strains when choosing the knee-point or the final setting time as the time-zero. On the other hand, choosing the initial setting time would lead to higher values of strain (Figure 11, Figure 12, Figure 13 and Figure 14). Similar results were found in literature for cement paste mixtures with and without shrinkage reducing admixtures [4]. It is important to highlight that, given the nature of the progressive cement hydration, the transition from a fluid to a solid state does not occur in an specific (discrete) point in time, but over several hours, and the use of test methods applied in this study provide a time within this transition period.

The initial setting time occurs before the knee-point, while the material is still more fluid than solid. At this moment, the shrinkage strain is still not dangerous to the material since there is not much development of stresses inside the pores. It is then reasonable to state that the choice of the initial setting time as time-zero leads to an overestimation of the effects of the shrinkage and an underestimation of the internal curing promoted by the SAPs.

### 3.5. Practical Issues

Even though a correlation was found between both techniques used to determine the final setting time (the Vicat method and the UPV) and no relevant difference was observed in the autogenous strain when choosing the final setting time or the knee-point as the time-zero, some practical issues should be taken into account when making a choice between those methods.

As stated before, both the Vicat method and the UPV are based on two different physical principles. While the first one relies on the development of the mechanical strength of the material to resist the penetration of the test needle, the UPV is more related to the development of the microstructure of the material. 

The conditions of the both tests (exposition to the environment temperature and humidity and volume of the specimen) are different from each other, which at some point could lead to very different results. When using either of them to determine the time-zero, the conditions of the test should be as close as possible to the ones in the test method used to determine the autogenous strain. Not only that, the specimens should be prepared from the same mixture batch and start at the same time, which can be labor intensive.

The measurements of autogenous shrinkage performed in accordance to the Standard ASTM C1698–09 [48] relies on a specimen in a closed tube where no (or almost no) exchange of moisture is expected, which is impossible to be guaranteed with the automated Vicat apparatus, for example.

By relying only on the autogenous strain test method to determine the time-zero, not only time, but material can be saved and the test executor can be assured that both the autogenous strain and the time-zero will be determined from a mixture under the same environmental conditions and geometric characteristics.

## 4. Conclusions

In this paper, different methods for assessing the time-zero were evaluated for mixtures with and without superabsorbent polymers as internal curing agents. Even though the different tests are related to different physical phenomena taking place in the cement paste mixtures, the specimens have different geometries and each test has its own artefacts, the following can be concluded:

- There is a correspondence between the initial and the final setting times determined by the Vicat test and the UPV measurements.
- A transition point representing the moment when the fluid material starts to develop a solid skeleton can easily be determined based on the strain rate from the autogenous shrinkage test. This is the so-called knee-point.
- There is a correspondence between the transition marked by the knee-point and the highest build-up in the capillary pressure for the mixtures with effective water-to-cement ratio of 0.30. As the effective water-to-cement ratio goes higher, the highest build-up in the capillary pressure occurs closer to the final setting time which is later in time in comparison with the moment of transition marked by the knee-point.
- The addition of SAPs causes a delay in the moment of transition and build-up in capillary pressure, showing internal curing abilities.
- Choosing the time-zero as the initial setting time is not suitable and leads to an overestimation of the shrinkage strain and an underestimation of the internal curing effect promoted by the SAPs.
- There is not a relevant difference in choosing the time zero as the knee-point or the final setting time. However, choosing the knee-point seems more suitable since it can easily be determined on the results from the autogenous shrinkage test. No other additional test is then needed for determining the time-zero, which can save time and material.

## Figures and Tables

**Table 1:** Composition of the cement pastes, showing the effective (–), the entrained (–) and the total water-to-cement ratio (–), with the amount of SAPs (m%), the amount of superplasticizer (m%) used and the slump flow of the mixtures measured 10 min after the first contact with water (mm).

Mixture	Effective w/c	Additional w/c	Total w/c	Amount of SAPs (m%) ^1^	Amount of Superplasticizer (m%) ^1^	Slump Flow after 10 min (mm, n = 3)
REF0.3	0.30	0	0.30	0	0.30	29.50 ± 0.50
REF0.354	0.354	0	0.354	0	0.30	43.25 ± 0.35
SAP1	0.30	0.054	0.354	0.20	0.30	26.75 ± 0.35
SAP2	0.30	0.054	0.354	0.25	0.30	29.83 ± 0.28

^1^ m% versus cement.

**Table 2:** Average values and standard deviation of the initial and final setting time determined by the Vicat test and UPV (n = 2).

Mixture	Initial Setting [h]		Final Setting [h]	
	Vicat	UPV	Vicat	UPV
REF0.3	5.34 ± 0.53	5.00 ± 0.47	11.38 ± 0.18	10.34 ± 0.47
REF0.354	6.88 ± 1.24	5.67 ± 0.47	13.13 ± 0.53	12.34 ± 0.47
SAP1	6.63 ± 0.53	6.67 ± 0.94	12.63 ± 1.24	11.67 ± 0.47
SAP2	6.25 ± 0.00	7.25 ± 1.30	12.25 ± 0.71	13.21 ± 0.65

**Table 3:** Knee-point values for all mixtures (n = 2)

Mixture	Knee-Point (h)
REF0.3	9.85 ± 0.66
REF0.354	9.89 ± 0.65
SAP1	13.78 ± 0.29
SAP2	11.21 ± 0.81
